# Gut Microbiome Composition and Metabolic Capacity Differ by *FUT2* Secretor Status in Exclusively Breastfed Infants †

**DOI:** 10.3390/nu15020471

**Published:** 2023-01-16

**Authors:** Alexander W. Thorman, Grace Adkins, Shannon C. Conrey, Allison R. Burrell, Ying Yu, Brendon White, Rachel Burke, David Haslam, Daniel C. Payne, Mary A. Staat, Ardythe L. Morrow, David S. Newburg

**Affiliations:** 1 Department of Environmental and Public Health Sciences, Division of Epidemiology, University of Cincinnati College of Medicine, Cincinnati, OH 45220, USA; 2 St. Jude’s Graduate School of Biomedical Sciences, Memphis, TN 38105, USA; 3 Department of Pediatrics, Division of Infectious Diseases, University of Cincinnati College of Medicine, Cincinnati Children’s Hospital Medical Center, Cincinnati, OH 45220, USA; 4 Department of Pharmaceutical Sciences, College of Pharmacy, The University of Tennessee Health Science Center, Memphis, TN 38163, USA; 5 Division of Viral Diseases, Centers for Disease Control and Prevention, Atlanta, GA 30333, USA

**Keywords:** *FUT2*, secretor status, breastfed, microbiome

## Abstract

A major polymorphism in the fucosyltransferase2 (*FUT2*) gene influences risk of multiple gut diseases, but its impact on the microbiome of breastfed infants was unknown. In individuals with an active *FUT2* enzyme (“secretors”), the intestinal mucosa is abundantly fucosylated, providing mutualist bacteria with a rich endogenous source of fucose. Non-secretors comprise approximately one-fifth of the population, and they lack the ability to create this enzyme. Similarly, maternal secretor status influences the abundance of a breastfeeding mother’s fucosylated milk oligosaccharides. We compared the impact of maternal secretor status, measured by *FUT2* genotype, and infant secretor status, measured by *FUT2* genotype and phenotype, on early infant fecal microbiome samples collected from 2-month-old exclusively breastfed infants (*n* = 59). Infant secretor status (19% non-secretor, 25% low-secretor, and 56% full-secretor) was more strongly associated with the infant microbiome than it was with the maternal *FUT2* genotype. Alpha diversity was greater in the full-secretors than in the low- or non-secretor infants (*p* = 0.049). Three distinct microbial enterotypes corresponded to infant secretor phenotype (*p* = 0.022) and to the dominance of *Bifidobacterium breve*, *B. longum*, or neither (*p* < 0.001). Infant secretor status was also associated with microbial metabolic capacity, specifically, bioenergetics pathways. We concluded that in exclusively breastfed infants, infant—but not maternal—secretor status is associated with infant microbial colonization and metabolic capacity.

## 1. Introduction

At birth, the infant gut is colonized sparsely, if at all. The first significant introduction of microbes to the neonate results from vertical transmission from the mother to infant, seeding the infant gut with maternal bacteria [1]. Initially, a mixture of aerobes and facultative anaerobes thrive in the oxygenated lumen of the gut. However, as the newborn matures and oxygen tension decreases, the microbial community shifts to being comprised of facultative and obligate anaerobes [2] and the genus *Bifidobacterium* generally predominates [3].

Feeding infants with human milk strongly influences infant microbial colonization and selectively supports the growth of *Bifidobacterium*, as has been shown in large multi-site studies [4,5]. A key mediator of the effects of human milk on the infant microbiota is the oligosaccharide fraction [3]. Human milk oligosaccharides (HMOS) are short polysaccharides whose base structure is lactose (Galβ1-4Glc). Their prebiotic characteristics strongly influence microbial colonization and metabolism. While human intestinal digestive enzymes do not hydrolyze the glycosidic bonds of HMOS [6], gene clusters of *Bifidobacterium* spp. are devoted to the digestion of HMOS [2,7]. Bifidobacterial species vary in their enzyme repertoire for consuming HMOS and other carbohydrates [8,9]. The digestion of HMOS by bifidobacterium and other beneficial bacteria produces primarily short chain fatty acids and dicarboxylic acids that serve as metabolic intermediates and control elements in microbial and host metabolism.

**Figure 1:**
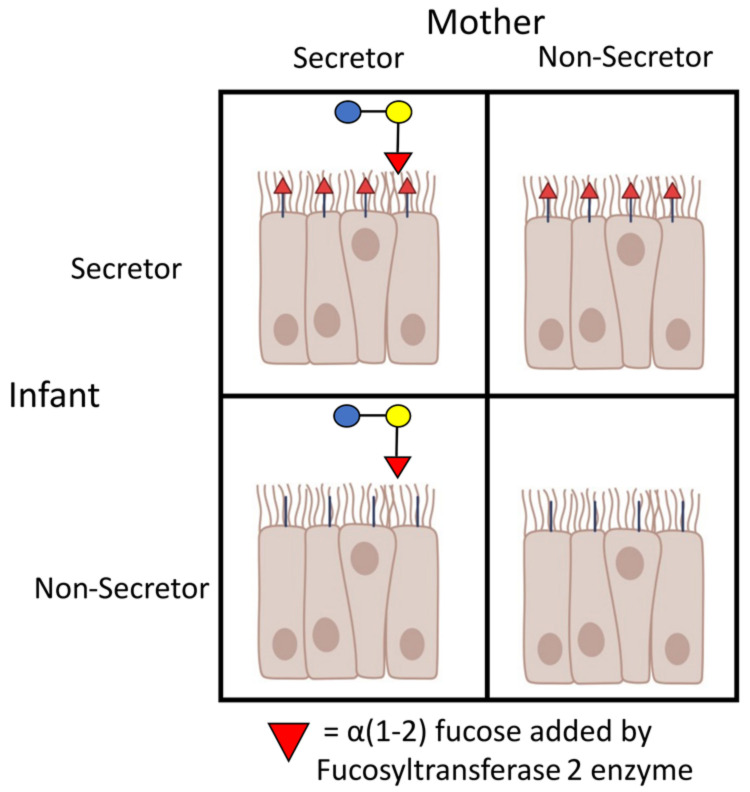
Potential contributions of fucose to the intestinal mucosa of breastfeeding infants by the *FUT2* gene product in the infant gut and by a secretor’s milk.

The quantity and composition of HMOS can range from 5 to 12 g/L in a mothers’ milk [10,11], depending on the stage of lactation, maternal microbial colonization [12], and maternal genetics [13,14]. *FUT2*, sometimes referred to as *Se2*, encodes α1,2-fucosyltransferase II and controls the presence or absence of water-soluble blood group antigens in bodily fluids (secretor status) such as saliva, tears, urine, semen, and breast milk in an autosomal dominant manner. Only secretor women can synthesize the α1,2-fucose-containing oligosaccharides which generally account for 25–50% of the HMOS in human milk. Non-secretor individuals comprise 20% of the United States’ population. Mothers who are non-secretors not only lack a class of fucosylated HMOS, but they also have a reduced oligosaccharide content in their milk. The presence or absence of the *FUT2* enzyme also controls this same carbohydrate motif in the human gut, affecting the fucosylation of mucosal glycans (Figure 1). In particular, α1,2-fucosyltransferase II catalyzes the transfer of L-fucose from a guanosine diphosphate-β-L-fucose to the terminal galactose of O- and N-linked oligosaccharide chains of glycoproteins and glycolipids at the cell surface. α1,2-fucosyltransferase II preferentially fucosylates glycolipids in the antrum, cecum, and colon, producing epitopes that are important in the regulation of the cell surface expression of glycoconjugates, cell proliferation, cell-cell interactions, and host-microbiota interactions [15,16,17]. In the absence of a functional *FUT2* gene product in a non-secretor infant, mucosal surfaces of the gut are poorly fucosylated. To some extent, *Bifidobacterium* spp. can utilize fucosylated and other oligosaccharides harvested from the human gut [9,15], but this is typically a secondary source via cross-feeding initiated by organisms that are more efficient at harvesting oligosaccharides from the gut mucin layer [16,17].

The *FUT2* polymorphism has been shown to influence the risk of infectious and inflammatory diseases. For example, inflammatory bowel disease, peptic ulcer disease, and other gastrointestinal disorders [18,19,20,21]; type 1 diabetes [22]; and a reduced response to rotavirus vaccine [23] are each increased in non-secretor individuals, whereas the risk of critical COVID-19 [24], norovirus, and rotavirus [25,26,27] are increased in secretor individuals. As a result, understanding the role of the *FUT2* gene in shaping the host microbiota is of global interest [21,28]. However, only a few studies that incorporate the status of both mother and child and that focus on the microbiota and the *FUT2* polymorphism have been conducted in breastfed infants [29]. The exclusively breastfed infant presents a particular case of dual (dietary and endogenous) fucose sources (Figure 1). However, studies on the breastfed infant have typically focused entirely on the secretor type of the mother’s milk alone, leaving the contribution of the infant’s own gut fucosylation undefined. We hypothesized that the fucosylated oligosaccharides of both a mother and an infant contribute to a healthy infant gut microbiota. Therefore, the studies described herein were designed to compare the relative contribution of *FUT2* status of both the mother and infant in defining the microbiome and microbial metabolic capacity of exclusively breastfed 2-month-old infants.

## 2. Methods

For the current analysis, we utilized a subset of data and samples collected as part of the CDC-sponsored PREVAIL (Pediatric Respiratory and Enteric Virus Acquisition and Immunogenesis Longitudinal) birth cohort, conducted in Cincinnati, Ohio from 2017 to 2020. PREVAIL included only mothers who were generally healthy, were enrolled in the last trimester of pregnancy, had a singleton birth, and had no history of congenital anomalies, HIV, or illicit drug use. A detailed description of the cohort has previously been published [30]. This study was approved by the institutional review boards at the CDC, Cincinnati Children’s Hospital Medical Center, and the enrolling birth hospitals.

The current analysis was restricted to only those infants who had a stool sample collected between 6 and 9 weeks postpartum and were exclusively breastfed at the time of sample collection. Samples were collected throughout the first two years of life, and the present study focused on the saliva collection used for genotyping and secretor phenotyping and the stool and demographics data from the infants between 6 and 9 weeks of age. Saliva samples were collected from study mothers and infants for DNA testing using DNA Genotek kits. A Salimetrics kit was used for an additional infant saliva collection between 12 and 24 months of age for the purposes of secretor phenotyping. Infant stool samples were collected from study mothers via courier in coolers, aliquoted, and stored at −80 °C prior to DNA isolation.

Questionnaires were administered to the study mothers at the time of enrollment into the study to characterize their sociodemographics and health history, with additional questionnaires administered every few months at in-person study visits or via phone calls or text questionnaires to update the mother–infant breastfeeding status (Table 1). Exclusive breastfeeding was defined as feeding the infant only mother’s milk, with no other source of food.

### 2.1. Measurement of Secretor Status

PCRs of both the maternal and infant saliva samples defined the secretor genotypes of the subjects; a 428 G > A point mutation that results in a premature stop codon was used to identify those who lacked the functional *FUT2* gene [31]. Following previously published methods [23,32], the infant saliva samples were analyzed for the secretor phenotype by ELISA using the UEA-1 lectin method, which reliably exhibits specific binding to α-1,2-linked fucose, which is the secretor motif (referred to as the H-antigen).

Analysis of the H-antigen was conducted as follows: Saliva samples were diluted 200-fold and coated onto 96-well microtiter plates at 4 °C overnight. The following day, the plates were blocked with 5% BSA and assayed using HRP-conjugated UEA-I (EYlabs) lectin. Using published cut-points [23], genetically *FUT2*-positive infants (GA or GG genotypes) were defined as low-secretors if they were had an EIA optical density (OD) value that was <0.35. Infants with UEA-1 values of ≥0.35 were considered to be full-secretors.

### 2.2. Metagenomics Workflow

DNA was extracted from the raw stool samples in cryogenic storage that had sufficient sample volume. Extraction was performed from 0.25 g of each stool sample with a Power Fecal DNA Isolation Kit^®^ (MO BIO^®^ (Carlsbad, CA, USA)), per the kit instructions. The DNA concentration was measured using Qubit^®^. Nextera XT^®^ adapters (Illumina Corp. (San Diego, CA, USA)), which generated an amplified library that was sequenced to obtain 150 bp DNA paired-end reads to a depth of 10 million base pairs per sample using an Illumina NovaSeq 6000 (Illumina Corp. (San Diego, CA, USA)). The reads were mapped to bacterial species using Kraken2/Bracken [33,34] and to gene pathways using HUMAnN2 [35].

### 2.3. Statistical Analyses

Statistical analyses were performed using ASV tables generated through the outputs of Kraken2/Bracken [33,34] or HUMAnN2 [35] after converting the read counts to relative abundance. Alpha- and beta-diversity were determined using the vegan package, and LEfSe was determined by using the MicrobiomeMarker [36] package in R. Alpha-diversity was calculated as Shannon diversity and beta-diversity was calculated as Bray–Curtis distance.

## 3. Results

Among the 245 mother–infant pairs enrolled in PREVAIL, 211 had an infant stool sample collected at 6 to 9 weeks. Of these, 59 (28%) mother–infant pairs were exclusively breastfeeding at the time of stool sample collection and were included in this analysis. The majority of the mothers in the study were educated beyond high school (85%), were white (86%), had given birth by vaginal delivery (71%), and were married or partnered (88%), and half of the mothers had been treated with antibiotics during the intrapartum period (Table 1). The secretor status of the study infants was statistically different by maternal secretor status, as would be expected based on Mendelian inheritance. The secretor status of the study infants did not differ by the measured demographics except for maternal education, which was not expected and may have occurred by chance.

Of the 59 mothers included in this analysis, 57 (97%) had their DNA successfully genotyped; 9 (16%) were non-secretors (*FUT2*-negative, with an AA genotype) and 48 (84%) were secretors (*FUT2*-positive, with GA or GG genotypes). Of the study infants, 58 (98%) had their DNA successfully genotyped; the single infant missing its genotype had a saliva EIA result that was consistent with being a secretor, which was used to impute the missing genetic status. This approach identified 11 (19%) infants as genetic non-secretors and 48 (81%) as secretors.

Five of the infants lacked the saliva for the analysis of secretor phenotype; of these, all were genetic secretors. Of the 43 secretors with phenotype results, we found 15 (35%) low-secretors and 28 (65%) full-secretors. No maternal or infant characteristics, including the GA or GG genotypes, were associated with the UEA-1 OD values of the secretor infants. Lacking any other information to guide imputation, the five infants lacking phenotype results, but who were known to be genetic secretors, were imputed as the dominant secretor group (full-secretors).

**Figure 2:**
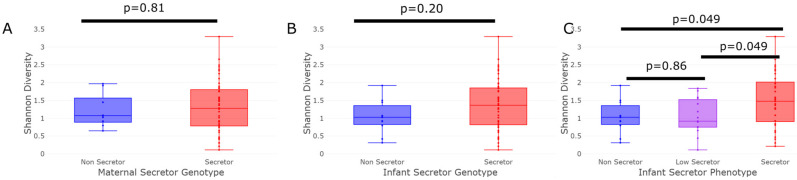
Alpha diversity by secretor status. The presence of secretor (presence/absence of functional *FUT2* gene product) milk from a mother was not accompanied by significant changes in the overall alpha-diversity of an infant’s gut microbiota (**A**) (Kruskal–Wallis *p* = 0.81). The secretor genotype of the infants was not significantly associated with a change in the alpha-diversity of an infant’s gut (**B**) (Kruskal–Wallis *p* = 0.20). However, the secretor phenotype (measured as the presence of the H-antigen blood group antigens in the saliva) of the infants revealed no significant differences in the alpha diversity measured as the Shannon index between the low- and non-secretors (Kruskal–Wallis *p* = 0.86), but the Shannon index of the full-secretor group was significantly higher than that of the low- and non-secretors (**C**) (Kruskal–Wallis *p* = 0.049).

The alpha-diversity of the infant fecal samples did not differ significantly by *FUT2* genotype for either the mother (Figure 2A) or the infant (Figure 2B). However, by secretor phenotype, the full-secretors exhibited a significantly (*p* = 0.049) higher level of microbial diversity than the non- and low-secretors (Figure 2C). The Shannon diversity of the non-secretor and low-secretor infants did not differ significantly.

**Figure 3:**
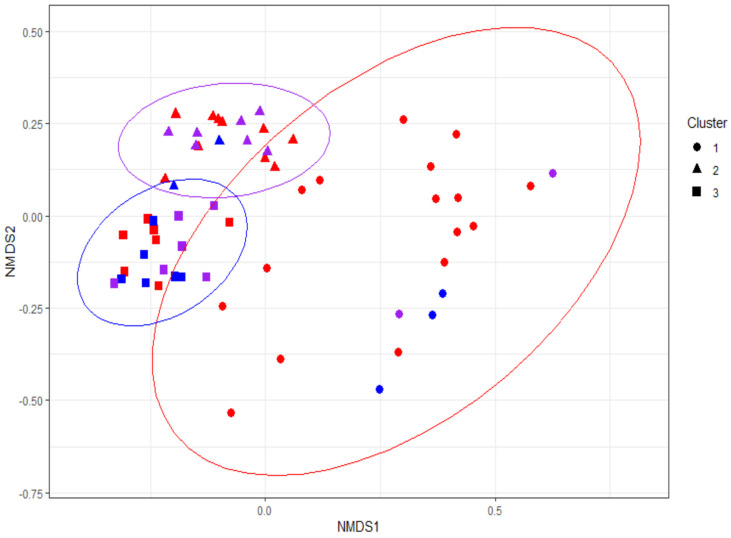
Beta-diversity by infant secretor phenotype. The Bray–Curtis distance between the subjects resulted in significant clustering by infant secretor status by infant phenotype (Adonis *p* = 0.043). The three clusters generated by the k-means are depicted as triangles, circles, and squares, and the ellipses correspond to the k-means (k = 3) clusters of all points, irrespective of phenotype. A significant association of phenotype to these clusters occurred. Thus, at least three distinct enterotypes of microbial communities could be identified and are significantly associated with infant secretor phenotype.

The microbial beta-diversity was measured by Bray–Curtis dissimilarity. Infant gut microbial diversity did not differ by maternal secretor status (Supplemental Figure S1A). However, beta-diversity was significantly associated with both infant secretor genotype (Supplemental Figure S1B, *p* = 0.025) and phenotype (Figure 3, *p* = 0.043). Thus, contrary to maternal *FUT2* genotype, we found that infant secretor status was significantly associated with the overall composition of an infant’s gut.

**Figure 4:**
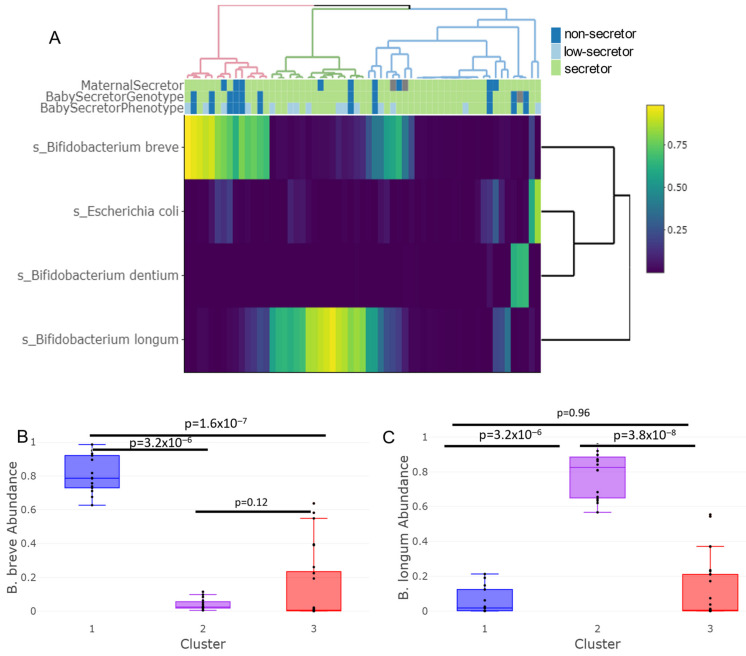
Heatmap of the highest variance in species across all subjects. A high abundance of *Bifidobacterium* occurred regardless of secretor status, but the species of *Bifidobacterium* varied. (**A**) By hierarchical clustering, significant clustering occurred in the infant secretor genotype (*p* = 0.030) and phenotype (*p* = 0.012) but not in the maternal secretor genotype (*p* = 0.80). (**B**) The cluster associated with non-secretors was enriched for *Bifidobacterium breve* by infant genotype and phenotype (*p* = 7.9 × 10^−8^). (**C**) *Bifidobacterium longum* was enriched in the cluster primarily associated with the low-secretors (*p* = 3.4 × 10^−8^). Additionally, there were some subjects that demonstrated a microbiota not dominated by these two species of *Bifidobacteria* that were primarily associated with the full-secretor population.

To examine the differences in relative abundance, the ten species with the highest variation across all samples were considered, after which six species were excluded due to a lack of sufficient abundance, and four were sufficiently abundant to be maintained in the analysis (>15% of the total abundance in at least 5% of the samples). These four species were then hierarchically clustered by subject (Figure 4A), which resulted in the identification of three distinct clusters that generally corresponded to the three clusters identified in Figure 3 by the k-means. These clusters differed in their composition of infant secretor genotype (Fisher’s exact test *p* = 0.030) and infant phenotype (Fisher’s exact test *p* = 0.012) but not by maternal genotype (Fisher’s exact test *p* = 0.80). The left-most cluster highlighted in red (cluster 1), which was driven by a predominance of *Bifidobacterium breve*, roughly corresponds to the blue cluster in Figure 3. The infant non-secretors were most prevalent within cluster 1 (six non-secretors, four low-secretors, and four full-secretors). The middle cluster highlighted in green (cluster 2), driven by a predominance of *Bifidobacterium longum*, roughly corresponds to the purple cluster in Figure 3. The right-most cluster highlighted in blue (cluster 3) is composed of several other species and genera and corresponds roughly with the red cluster in Figure 3. The secretor genotype was distributed across cluster 2 (one non-secretor, seven low secretors, and eight full-secretors) and cluster 3 (4 non-secretors, 4 low-secretors, and 24 full-secretors); however, when accounting for secretor phenotype, cluster 2 was enriched in low-secretors. This supports the concept that the low-secretor phenotype may be distinct from both full-secretors and non-secretors.

To provide greater detail regarding the observed microbial differences shown in Figure 4A between the clusters that corresponded to non-secretors, low-secretors, and full-secretors, the relative abundances of *B. breve* (Figure 4B) and *B. longum* were analyzed by cluster (Figure 4C). The cluster associated with non-secretor infants demonstrated a significant increase in *B. breve* abundance compared to the other clusters (Kruskal–Wallis *p* = 7.9 × 10^−8^) and the low-secretor-associated cluster had an increase in *B. longum* abundance (Kruskal–Wallis *p* = 3.4 × 10^−8^). For the abundance of *B. breve*, the full-secretor and non-secretor phenotypes displayed trends consistent with their respective genotypes (Kruskal–Wallis *p* = 0.12), whereas the low-secretors were indistinguishable from either group (Kruskal–Wallis *p* = 0.33, *p* = 0.42). For the abundance of *B. longum*, the full-secretor and non-secretor phenotypes also displayed trends consistent with their respective genotypes (Kruskal–Wallis *p* = 0.20). Among the low-secretors, the abundance of *B. longum* was greater than it was in microbiota of the non-secretors (Kruskal–Wallis *p* = 0.052). The relative abundance of the most abundant organisms in these three clusters is shown in Supplemental Figure S2. The data from this limited sample size are consistent with the low-secretors being distinct from both the non-secretors and the full-secretors in microbial composition.

**Figure 5:**
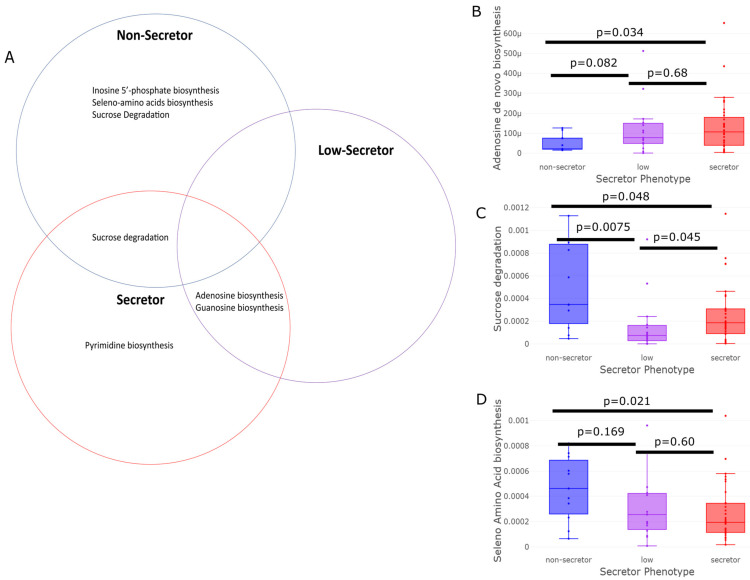
Pathway enrichment of metabolic capacity showing the differences between the secretor phenotypes. The metagenomic sequences were aligned to genes and pathways using HUMAnN2 and compared across the secretor phenotypes using LEfSe. (**A**) The pathways enriched in the full-secretors and low-secretors demonstrated a bioenergetics signature, while the non-secretors had more genes dedicated to the utilization of sucrose and the production of seleno-amino acids. The adenosine and guanosine biosynthesis pathways showed significant increases in the full-secretor individuals ((**B**), *p* = 0.034), whereas the sucrose degradation ((**C**), *p* = 0.0075) and seleno-amino acid biosynthesis pathways ((**D**), *p* = 0.021) were enriched in the microbiota of the non-secretor individuals.

The sequenced reads were mapped to gene pathways using HUMAnN2 and compared via LEfSe analysis. The relative abundance of each pathway identified by LEfSe was compared across the secretor groups (Figure 5). Phenotypically full-secretor individuals, compared to non-secretors, had microbiota enriched in bioenergetic pathways as evidenced by a significant increase in adenosine and guanosine de novo biosynthesis (Figure 5B, *p* = 0.034); this trend was also evident in the low-secretors relative to the non-secretors (*p* = 0.089). Non-secretors were, instead, enriched in their ability to biosynthesize seleno-amino acids relative to the full-secretors (Figure 5C, *p* = 0.021). Both the full-secretor (*p* = 0.045) and non-secretor (*p* = 0.0075) microbiota were significantly enriched in genes associated with the degradation of sucrose compared to that of the low-secretors; however, the non-secretor microbes were even further enriched in sucrose degradation genes compared to the secretors (Figure 5B, *p* = 0.048).

Taken together, these data indicate that the phenotype of a low-secretor in infants was associated with a distinct microbial enterotype enriched in *B. longum* but not necessarily other *Bifidobacteria*.

## 4. Discussion

Fifty-nine mother–infant pairs from the PREVAIL cohort in Cincinnati, Ohio who were exclusively breastfeeding at 2 months of age were studied to focus on the relative contribution of maternal and infant *FUT2* status to the infant microbiome. Nutrition studies have typically overlooked infant secretor status, focusing solely on the milk type of the mother. Notably, the study reported herein found that the *FUT2* status of an infant, but not a mother’s *FUT2* genotype, is a significant determinant of the infant microbiome in exclusively breast-fed infants.

In this study, the microbiomes of exclusively breastfed children were separated into three microbial clusters, or enterotypes, that were significantly associated with infant secretor status and bifidobacterial profile, as well as microbial metabolic capacity. Differences by infant genotype alone were detected by the analysis of beta-diversity (Figure 3) and hierarchical clustering (Figure 4). No significant association was apparent between *FUT2* genotype and the relative abundance of *Bifidobacterium* as a genus. Nevertheless, the microbiota of non-secretor infants formed a significantly distinct cluster (*p* = 0.030), and this first cluster was enriched in the species *B. breve*. *FUT2* genotypes also differed by the metabolic capacities of their gut microbiota. The microbiota of secretor infants had higher levels of genes related to adenosine and guanosine de novo biosynthesis, while the microbiota of non-secretors had higher levels of genes related to the degradation of sucrose and the biosynthesis of seleno-amino acids.

Phenotyping genetic secretors as either low- or full-secretors was associated with even more pronounced differences in the infant microbiome. The microbiota of the full-secretors had significantly greater alpha-diversity than that of low-secretors or non-secretors. Further, the microbiota of low-secretors were associated with another distinct microbial cluster that was enriched in *B. longum*. While secretor status was significantly associated with the clusters enriched in *B. longum* or *B. breve*, there was not a direct relationship between these organisms and secretor phenotype.

The cluster associated with full-secretors had microbiomes rich in either a combination of *B. breve* and *B. longum* or microbiomes with virtually no *Bifidobacteria*. While we were unable to identify the subspecies of *Bifidobacteria*, prior work using samples from the same cohort confirmed that *B. breve* and *B. longum* were predominant and that the *B. longum* were nearly all *B. longum* subsp. *longum,* with scant colonization by *B. longum* subsp. *Infantis* [5]. We note that *B. breve* and *B. longum* subsp. *longum* are often found in adults, and unlike *B. longum* subsp. *infantis*, they are not specialized to use HMOS [9].

Several of our novel findings require careful consideration in light of prior studies. All mother–infant pairs in this analysis were healthy and exclusively breastfed at 2 months, consistent with global and U.S. public health recommendations. The lack of an association between the infant microbiota and maternal *FUT2* genotype at two months of lactation suggests that the oligosaccharides provided by the mother were sufficiently equivalent or were overshadowed by the glycans provided by the gut mucosa of the infant. The finding that infant *FUT2* status significantly impacted their microbiome is consistent with other studies on the *FUT2* genotype and microbiome, which have typically been conducted in adult populations, including studies on healthy individuals and studies on gut and respiratory diseases [21,28,29,36]. However, the literature on *FUT2* and the microbiome remains controversial, as diverse, large-scale studies have reported differing results [22]. Variability in the literature regarding *FUT2* and the microbiome may well be due to study population differences in diet, host health status, medication use, and microbial and other exposures. Future investigations into the relationship between *FUT2* and the microbiome that address these important modifiers are warranted.

*FUT2* provides an interface between the host and the microbial world. Its expression is induced by early colonization via the ERK and JNK signaling pathways [37], and it is upregulated or downregulated by antibiotic use, microbial mutualists, and pathogens [38]. To avoid strong influences that are not well understood and that cannot be controlled, we limited our study to exclusively breastfed infants from a single cohort. The results of this study add intriguing evidence that *FUT2* status can influence or be influenced by microbial colonization, and our findings are consistent with a prior report on infants [32] where “low” or “partial” *FUT2* phenotypic expression may have been important to differentiate.

Prior to the routine access to *FUT2* genotyping, secretor status was routinely measured by measuring the H-antigen in saliva. As phenotyping alone cannot distinguish low-secretors from non-secretors, there were more non-secretors identified than would now be found by genotyping [39]. There is currently no universally accepted method for phenotyping secretor status. The phenotyping assays used herein found that all non-secretors had very low salivary H-antigen, but without genotyping data, the phenotyping criteria alone would have misclassified many low-secretors as non-secretors. Nevertheless, in this study, the low-secretor phenotype of genetic secretors provided additional insight into microbial colonization. Therefore, we propose that the best approach to studying *FUT2* functions is to measure genotype and to further characterize genetic secretors into those with low and abundant H-antigen expression.

In this study of PREVAIL infants, 19% of the infants were genetic non-secretors, consistent with expectations [40]. In the United States and the PREVAIL cohort, the non-secretor state results from a single-nucleotide polymorphism (SNP rs601338), G- > A at position 428 [41], which is common in populations originating from Europe, Middle East- North Africa, and Sub-Saharan Africa [41]. The non-secretor phenotype occurs with similar frequency in East Asia due to a different SNP (rs1047781) [42].

There were several limitations in our study. This was a single-site study with a modest sample size. Further work is required to analyze this newly revealed relationship between *FUT2* status and the microbiome in exclusively breastfed infants compared to those with other feeding regimens. The HMOS content of a mothers’ milk, not analyzed for this study, could also contribute toward modulating the infant microbiota and add to the interpretation of the current data. The strengths of this study include its use of a widely accepted genotyping approach combined with secretor phenotyping which followed a previously published method with validated pre-established cut-points. Whole genome shotgun metagenomics provided the ability to accurately identify microbial species in the microbiome and to characterize the metabolic capacity of the microbiota.

The data presented herein, in conjunction with the deepening evidence of *FUT2*’s association with infectious and inflammatory conditions, indicate that continued research on *FUT2*’s interaction with the microbiota, microbial succession, microbial function, and infant gut health is important. For epidemiologic studies or trials, the concurrent use of genotyping and phenotyping appears to be critical for advancing the understanding of the microbiome associated with *FUT2* status. Further study of the impact of *FUT2* on microbial and host metabolism and immunomodulation will be important for understanding health conditions associated with dysbiosis.

## Figures and Tables

**Table 1:** Demographics of the exclusively breastfeeding dyads of the PREVAIL cohort.

Demographic Variable	Description	Non-Secretor	Low-Secretor	Full-Secretor	Total	Fisher’s Exact *p*-Value
Ethnicity	Hispanic	0 (0%)	0 (0%)	1 (3%)	1 (2%)	1
	Non-Hispanic	11 (100%)	15 (100%)	32 (97%)	58 (98%)	
Maternal Race	Black	3 (27%)	1 (7%)	4 (12%)	8 (14%)	0.34
	White	8 (73%)	14 (93%)	29 (88%)	51 (86%)	
Maternal Education	≤High School	5 (45%)	1 (7%)	3 (9%)	9 (15%)	0.016
	College/Trade	6 (55%)	14 (93%)	30 (91%)	50 (85%)	
Marital Status	Partnered	9 (81%)	15 (100%)	28 (85%)	52 (88%)	0.22
	Single	2 (19%)	0 (0%)	5 (15%)	7 (12%)	
Maternal Obesity	Yes	2 (19%)	2 (13%)	9 (27%)	13 (22%)	0.63
	No	9 (81%)	13 (87%)	24 (73%)	46 (78%)	
Delivery Mode	C-Section	5 (45%)	2 (13%)	10 (30%)	17 (29%)	0.17
	Vaginal Birth	6 (55%)	13 (87%)	23 (70%)	42 (71%)	
Maternal Secretor Status	Secretor	6 (55%)	13 (87%)	29 (88%)	48 (81%)	0.019
	Non-Secretor	5 (45%)	1 (7%)	3 (9%)	9 (15%)	
Intrapartum Antibiotics	Antbiotic Use	8 (73%)	7 (47%)	17 (52%)	32 (54%)	0.41
	No Antibiotic Use	3 (27%)	8 (53%)	16 (48%)	27 (46%)	
Total	Infant Secretor Status	11	15	33	59	

## Data Availability

The raw sequencing data are available at the NCBI SRA.

## Supplementary Materials

The following supporting information can be downloaded at: https://www.mdpi.com/article/10.3390/nu15020471/s1, Figure S1: Beta diversity ordinations of maternal genotype (**A**), infant genotype (**B**), and infant phenotype (**C**). Bray-Curtis dissimilarity between stool microbiota of breastfed infants does not significantly cluster by maternal secretor status ((**A**), Adonis *p* = 0.68). Bray-Curtis distance between subjects resulted in significant clustering by infant secretor status by both genotype ((**B**), Adonis *p* = 0.025) and phenotype ((**C**), Adonis *p* = 0.043). The 3 clusters generated by k-means are depicted as triangles, circles and squares and ellipses correspond to k-means (k = 3) clusters of all points, irrespective of phenotype. Significant association of infant genotype and phenotype to these clusters occurs.; Figure S2: Relative abundance of the most prevalent species found in each cluster.

## References

[1] Gritz E.C., Bhandari V. (2015). Corrigendum: The Human Neonatal Gut Microbiome: A Brief Review. Front. Pediatr..

[2] Sanidad K.Z., Zeng M.Y. (2020). Neonatal gut microbiome and immunity. Curr. Opin. Microbiol..

[3] Yu Z.-T., Chen C., Newburg D.S. (2013). Utilization of major fucosylated and sialylated human milk oligosaccharides by isolated human gut microbes. Glycobiology.

[4] Stewart C.J., Ajami N.J., O’Brien J.L., Hutchinson D.S., Smith D.P., Wong M.C., Ross M.C., Lloyd R.E., Doddapaneni H., Metcalf G.A. (2018). Temporal development of the gut microbiome in early childhood from the TEDDY study. Nature.

[5] Taft D.H., Lewis Z.T., Nguyen N., Ho S., Masarweh C., Dunne-Castagna V., Tancredi D.J., Huda M.N., Stephensen C.B., Hinde K. (2022). *Bifidobacterium* Species Colonization in Infancy: A Global Cross-Sectional Comparison by Population History of Breastfeeding. Nutrients.

[6] Berg J.M., Tymoczko J.L., Stryer L. (2002). Complex Carbohydrates Are Formed by Linkage of Monosaccharides. Biochemistry.

[7] Bode L. (2012). Human milk oligosaccharides: Every baby needs a sugar mama. Glycobiology.

[8] Milani C., Lugli G.A., Duranti S., Turroni F., Mancabelli L., Ferrario C., Mangifesta M., Hevia A., Viappiani A., Scholz M. (2015). Bifidobacteria exhibit social behavior through carbohydrate resource sharing in the gut. Sci. Rep..

[9] Thomson P., Medina D.A., Garrido D. (2018). Human milk oligosaccharides and infant gut bifidobacteria: Molecular strategies for their utilization. Food Microbiol..

[10] Thurl S., Munzert M., Boehm G., Matthews C., Stahl B. (2017). Systematic review of the concentrations of oligosaccharides in human milk. Nutr. Rev..

[11] Vinjamuri A., Davis J.C.C., Totten S.M., Wu L.D., Klein L.D., Martin M., Quinn E.A., Scelza B., Breakey A., Gurven M. (2022). Human Milk Oligosaccharide Compositions Illustrate Global Variations in Early Nutrition. J. Nutr..

[12] Seppo A.E., Kukkonen A.K., Kuitunen M., Savilahti E., Yonemitsu C., Bode L., Järvinen K.M. (2019). Association of Maternal Probiotic Supplementation with Human Milk Oligosaccharide Composition. JAMA Pediatr..

[13] Chaturvedi P., Warren C.D., Altaye M., Morrow A.L., Ruiz-Palacios G., Pickering L.K., Newburg D.S. (2001). Fucosylated human milk oligosaccharides vary between individuals and over the course of lactation. Glycobiology.

[14] Williams J.E., McGuire M.K., Meehan C.L., McGuire M.A., Brooker S.L., Kamau-Mbuthia E.W., Kamundia E.W., Mbugua S., Moore S.E., Prentice A.M. (2021). Key genetic variants associated with variation of milk oligosaccharides from diverse human populations. Genomics.

[15] Wacklin P., Tuimala J., Nikkilä J., Tims S., Mäkivuokko H., Alakulppi N., Laine P., Rajilic-Stojanovic M., Paulin L., de Vos W.M. (2014). Faecal Microbiota Composition in Adults Is Associated with the FUT2 Gene Determining the Secretor Status. PLoS ONE.

[16] Singh R.P. (2019). Glycan utilisation system in Bacteroides and Bifidobacteria and their roles in gut stability and health. Appl. Microbiol. Biotechnol..

[17] De Vuyst L., Leroy F. (2011). Cross-feeding between bifidobacteria and butyrate-producing colon bacteria explains bifdobacterial competitiveness, butyrate production, and gas production. Int. J. Food Microbiol..

[18] McGovern D.P., Jones M.R., Taylor K.D., Marciante K., Yan X., Dubinsky M., Ippoliti A., Vasiliauskas E., Berel D., Derkowski C. (2010). Fucosyltransferase 2 (*FUT2*) non-secretor status is associated with Crohn’s disease. Hum. Mol. Genet..

[19] Wu Y., Murray G.K., Byrne E.M., Sidorenko J., Visscher P.M., Wray N.R. (2021). GWAS of peptic ulcer disease implicates Helicobacter pylori infection, other gastrointestinal disorders and depression. Nat. Commun..

[20] Ellinghaus D., Ellinghaus E., Nair R.P., Stuart P.E., Esko T., Metspalu A., Franke A. (2012). Combined analysis of genome-wide association studies for Crohn disease and psoriasis identifies seven shared susceptibility loci. Am. J. Hum. Genet..

[21] Lopera-Maya E.A., Kurilshikov A., van der Graaf A., Hu S., Andreu-Sánchez S., Chen L., Vila A.V., Gacesa R., Sinha T., Collij V. (2022). Effect of host genetics on the gut microbiome in 7738 participants of the Dutch Microbiome Project. Nat. Genet..

[22] Giampaoli O., Conta G., Calvani R., Miccheli A. (2020). Can the *FUT2* Non-secretor Phenotype Associated with Gut Microbiota Increase the Children Susceptibility for Type 1 Diabetes? A Mini Review. Front. Nutr..

[23] Armah G.E., Cortese M.M., Dennis F.E., Yu Y., Morrow A.L., McNeal M.M., Lewis K.D.C., Awuni D.A., Armachie J., Parashar U.D. (2018). Rotavirus Vaccine Take in Infants Is Associated with Secretor Status. J. Infect. Dis..

[24] Kousathanas A., Pairo-Castineira E., Rawlik K., Stuckey A., Odhams C.A., Walker S., Russell C.D., Malinauskas T., Wu Y., Millar J. (2022). Whole-genome sequencing reveals host factors underlying critical COVID-19. Nature.

[25] Payne D.C., Currier R.L., Staat M.A., Sahni L.C., Selvarangan R., Halasa N.B., Englund J.A., Weinberg G.A., Boom J.A., Szilagyi P.G. (2015). Epidemiologic Association Between FUT2 Secretor Status and Severe Rotavirus Gastroenteritis in Children in the United States. JAMA Pediatr..

[26] Kambhampati A., Payne D.C., Costantini V., Lopman B.A. (2015). Host Genetic Susceptibility to Enteric Viruses: A Systematic Review and Metaanalysis. Clin. Infect. Dis..

[27] Currier R.L., Payne D.C., Staat M.A., Selvarangan R., Shirley S.H., Halasa N., Boom J.A., Englund J.A., Szilagyi P.G., Harrison C.J. (2015). Innate Susceptibility to Norovirus Infections Influenced by *FUT2* Genotype in a United States Pediatric Population. Clin. Infect. Dis..

[28] Rausch P., Rehman A., Künzel S., Häsler R., Ott S.J., Schreiber S., Rosenstiel P., Franke A., Baines J.F. (2011). Colonic mucosa-associated microbiota is influenced by an interaction of Crohn disease and *FUT2* (*Secretor*) genotype. Proc. Natl. Acad. Sci. USA.

[29] Binia A., Siegwald L., Sultana S., Shevlyakova M., Lefebvre G., Foata F., Combremont S., Charpagne A., Vidal K., Sprenger N. (2021). The Influence of *FUT2* and *FUT3* Polymorphisms and Nasopharyngeal Microbiome on Respiratory Infections in Breastfed Bangladeshi Infants from the Microbiota and Health Study. mSphere.

[30] Morrow A.L., Staat M.A., DeFranco E.A., McNeal M.M., Cline A.R., Conrey S.C., Schlaudecker E.P., Piasecki A.M., Burke R.M., Niu L. (2021). Pediatric Respiratory and Enteric Virus Acquisition and Immunogenesis in US Mothers and Children Aged 0-2: PREVAIL Cohort Study. JMIR Res. Protoc..

[31] Wacklin P., Mäkivuokko H., Alakulppi N., Nikkilä J., Tenkanen H., Räbinä J., Partanen J., Aranko K., Mättö J. (2011). Secretor Genotype (FUT2 gene) Is Strongly Associated with the Composition of Bifidobacteria in the Human Intestine. PLoS ONE.

[32] Morrow A.L., Meinzen-Derr J., Huang P., Schibler K.R., Cahill T., Keddache M., Kallapur S.G., Newburg D.S., Tabangin M., Warner B.B. (2011). Fucosyltransferase 2 Non-Secretor and Low Secretor Status Predicts Severe Outcomes in Premature Infants. J. Pediatr..

[33] Wood D.E., Lu J., Langmead B. (2019). Improved metagenomic analysis with Kraken 2. Genome Biol..

[34] Lu J., Breitwieser F.P., Thielen P., Salzberg S.L. (2017). Bracken: Estimating species abundance in metagenomics data. PeerJ Comput. Sci..

[35] Franzosa E.A., McIver L.J., Rahnavard G., Thompson L.R., Schirmer M., Weingart G., Lipson K.S., Knight R., Caporaso J.G., Segata N. (2018). Species-level functional profiling of metagenomes and metatranscriptomes. Nat. Methods.

[36] Pan C., Ning Y., Jia Y., Cheng S., Wen Y., Yang X., Meng P., Li C., Zhang H., Chen Y. (2021). Transcriptome-wide association study identified candidate genes associated with gut microbiota. Gut Pathog..

[37] Meng D., Newburg D.S., Young C., Baker A., Tonkonogy S.L., Sartor R.B., Walker W.A., Nanthakumar N.N. (2007). Bacterial symbionts induce a *FUT2*-dependent fucosylated niche on colonic epithelium via ERK and JNK signaling. Am. J. Physiol. Liver Physiol..

[38] Goto Y., Uematsu S., Kiyono H. (2016). Epithelial glycosylation in gut homeostasis and inflammation. Nat. Immunol..

[39] Ishitoya S., Yamamoto S., Mitsumori K., Ogawa O., Terai A. (2002). Non-secretor status is associated with female acute uncomplicated pyelonephritis. BJU Int..

[40] Kelly R.J., Rouquier S., Giorgi D., Lennon G.G., Lowe J.B. (1995). Sequence and Expression of a Candidate for the Human Secretor Blood Group α(1,2)Fucosyltransferase Gene (*FUT2*) homozygosity for an enzyme-inactivating nonsense mutation commonly correlates with the non-secretor phenotype. J. Biol. Chem..

[41] Ferrer-Admetlla A., Sikora M., Laayouni H., Esteve A., Roubinet F., Blancher A., Calafell F., Bertranpetit J., Casals F. (2009). A Natural History of *FUT2* Polymorphism in Humans. Mol. Biol. Evol..

[42] Ye B.D., Kim B.M., Jung S., Lee H., Hong M., Kim K., Moon J.W., Baek J., Oh E.H., Hwang S.W. (2019). Association of *FUT2* and ABO with Crohn’s disease in Koreans. J. Gastroenterol. Hepatol..

