# Supplementary material for: Gut Microbiome Composition and Metabolic Capacity Differ by FUT2 Secretor Status in Exclusively Breastfed Infants
Source: Nutrients. 2023 Jan 16;15(2):471. doi: 10.3390/nu15020471 (PMC9866411; doi:10.3390/nu15020471)
Supplement: Supplementary file 1 [file nutrients-15-00471-s001.zip › nutrients-2053428-supplementary.pdf]

Supplemental Materials

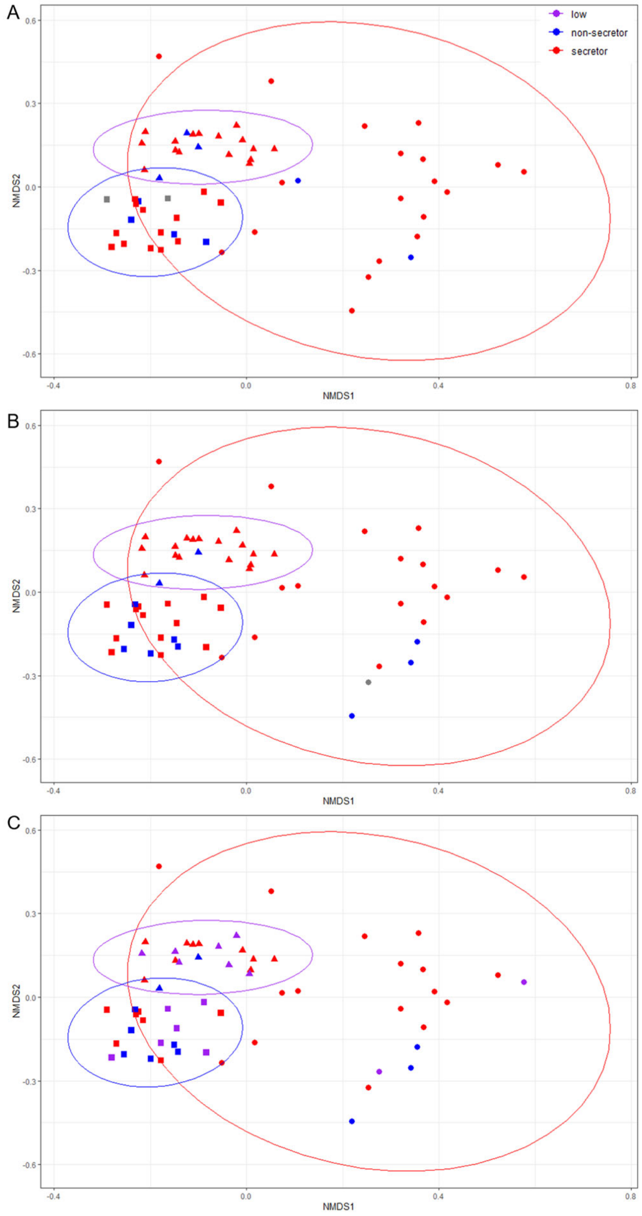

Figure S1. Beta diversity ordinations of maternal genotype (A), infant genotype (B), and infant phenotype (C). Bray-Curtis dissimilarity between stool microbiota of breastfed infants does not significantly cluster by maternal secretor status (**A**, Adonis p=0.68). Bray-Curtis distance between subjects resulted in significant clustering by infant secretor status by both genotype (**B**, Adonis p=0.025) and phenotype (**C**, Adonis p=0.043). The 3 clusters generated by k-means are depicted as triangles, circles and squares and ellipses correspond to k-means (k=3) clusters of all points, irrespective of phenotype. Significant association of infant genotype and phenotype to these clusters occurs.

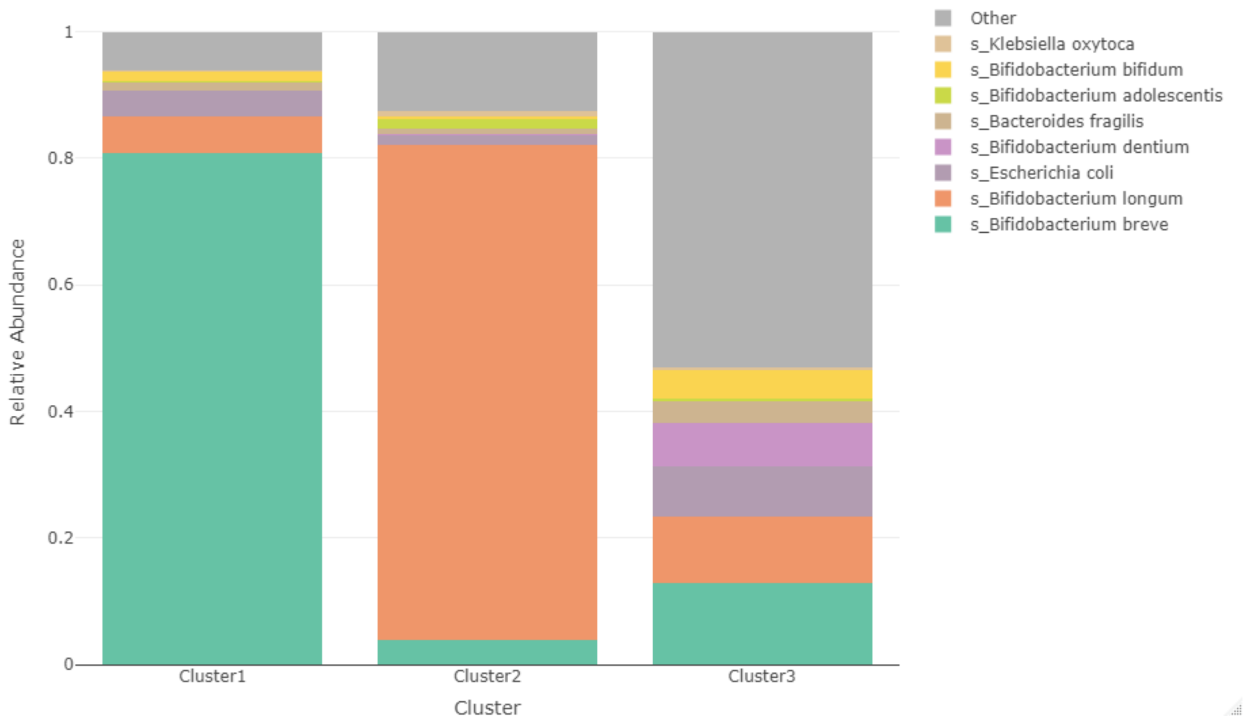

Figure S2. Relative abundance of the most prevalent species found in each cluster. The five highest mean abundance species from each cluster were compared across clusters. Cluster 1 was dominated by *B. breve*, while Cluster 2 was dominated by *B. longum*. Cluster 3 was poorly defined, consistent with the beta-diversity ordinations.
